# MiR-30c-5p/ATG5 Axis Regulates the Progression of Parkinson’s Disease

**DOI:** 10.3389/fncel.2021.644507

**Published:** 2021-05-25

**Authors:** Li Zhang, Xiufen Chen, Mingxiu Chang, Boning Jiao

**Affiliations:** Department of Neurology, The Fourth Hospital of Harbin Medical University, Harbin, China

**Keywords:** Parkinson’s disease, miR-30c-5p, autophagy-related (Atg) gene, apoptosis, autophagy

## Abstract

Serum miR-30c-5p correlates with Parkinson’s disease (PD), yet its role has not been illustrated. This research analyzed the function of miR-30c-5p in PD. The behavioral evaluation was performed on MPTP-treated PD mice transfected with miR-30c-5p agomiR, antagomiR, siATG5, or 3-MA (an autophagy inhibitor). Oxidative stress-related factors, miR-30c-5p, and apoptosis- and autophagy-associated proteins in brain tissues or cells were determined by molecular experiments. Tyrosine hydroxylase (TH) and dopamine metabolic markers were detected using immunofluorescence and Diode Array Detector (DAD), respectively. Effects of miR-30c-5p and its target gene Autophagy-related gene (ATG) 5 protein (ATG5) on MPP+-treated SH-SY5Y cells were determined through a series of molecular experiments. MiR-30c-5p was upregulated but ATG5 was downregulated in PD mice. MiR-30c-5p antagomiR attenuated the decrease of ATG5 in PD mice. MiR-30c-5p antagomiR partly alleviated the behavioral symptoms and inhibited the increases of malondialdehyde (MDA), catalase (CAT), and SOD in PD mice. The levels of Bcl-2, dopamine, dihydroxyphenylacetic acid (DOPAC), homovanillic acid (HVA), TH, and LC3 II were downregulated in PD mice, while Bax, cleaved caspase-3, P62, and LC3 I were upregulated. However, miR-30c-5p antagomiR partly reversed the levels of these factors in PD mice. 3-MA could block the effects of miR-30c-5p antagomiR on PD mice. MiR-30c-5p antagomiR attenuated apoptosis and induced autophagy in brain tissues of MPTP-treated mice by targeting ATG5. *In vitro* assay results also showed that silence of ATG5 reduced the protective effect of miR-30c-5p downregulation on the cells. MiR-30c-5p regulates the progression of Parkinson’s disease through attenuating ATG5-inhibited apoptosis and -induced autophagy.

## Introduction

Parkinson’s disease is a common age-related neurodegenerative disease mainly manifested by the loss of dopaminergic (DA) neurons in the substantia nigra (SN), and the accumulation of α-synuclein (α-syn) in the cytoplasm of neurons or glial cells (Lancet, 2017). The accumulation of α-syn in the neuropathological hallmark of Lewy bodies could induce the death of dopaminergic neurons (Ayers et al., 2018). Under normal conditions, the Lewy bodies will be degraded in dopaminergic cells, but in PD, dopaminergic cells are deficient in the degradation of damaged or abnormally modified proteins (Ebrahimi-Fakhari et al., 2012).

MicroRNAs (miRNAs) belong to non-coding RNA molecules with a length of 20–26 nucleotides, which negatively regulate gene expressions by binding to the 3’-untranslated regions (3’-UTRs) of specific mRNAs (Bartel, 2009). MiRNAs regulate cellular activity and control a range of physiological and pathological activations. In addition, evidence suggested that dysregulation of miRNAs occurs in neurodegenerative diseases. About 70% of miRNAs are expressed and regulated in the key signaling pathways in brain development (Nadim et al., 2017). MiR-30c was first identified in mouse heart and brain tissues (Lagos-Quintana et al., 2002). MiR-30c-5p could regulate inflammation (Ceolotto et al., 2017; Li et al., 2018), reactive oxygen species (Zou et al., 2017; Zhu et al., 2018; Wang L. et al., 2020), metabolism (Gao et al., 2020), and apoptosis (Du et al., 2017). Moreover, the abnormal elevation of miR-30c-5p has been reported in PD (Vallelunga et al., 2019). MiR-30c-5p may serve as a biomarker to improve the clinical diagnosis or prognosis prediction in patients with PD, and a potential therapeutic target for the treatment of PD.

Autophagy is a highly conserved homeostatic process widely present in eukaryotic cells, which is related to the destruction of unfolded proteins and damaged organelles (Rashid et al., 2015). Autophagy is inversely correlated with apoptosis induction in PD and plays a key role in PD. Restoring the balance between autophagy and apoptosis is a promising strategy for treating PD (Liu et al., 2019). Besides, autophagy can be considered as a process of self-recovery. The aggregation of misfolded and aggregated α-syn is involved in many neurodegenerative diseases such as PD against cell injury and maintains neuronal survival, and autophagy is the only mechanism for their clearance (Cook et al., 2012; Liu et al., 2019).

Autophagy-related gene (ATG) protein family plays a vital role in autophagy. So far, 40 ATG genes discovered are classified into five functional groups, among which autophagy-related gene 5 protein (ATG5), a protein of the ATG12 conjugation system, is specifically required for autophagy (Ye et al., 2018). ATG5 is located on human chromosome 6q21 and was originally identified in Burkitt’s lymphoma apoptotic cells (Otomo et al., 2013). ATG5-induced autophagy targets NF-κB signaling to limit epithelial inflammation after kidney injury (Peng et al., 2019). Autophagy is deficient in ATG5 −/− cells, accompanied by the increased production of reactive oxygen species (ROS; Xiong et al., 2015). Interestingly, miR-103a-3p regulates the progression of both autophagy and apoptosis of cardiomyocytes through binding to ATG5 (Zhang et al., 2019b). Previous research also showed that miR-30c-5p targets ATG5 to protect renal tubular epithelial cells against oxidative stress injury (Wang X. et al., 2020). Moreover, bioinformatics predicted that miR-30c-5p could target AGT5 in our preliminary study, indicating that miR-30c-5p has the potential of regulating autophagy. However, whether miR-30c-5p can affect the apoptosis and autophagy in PD through targeting ATG5 has not been reported yet.

Based on the research above, we were interested in investigating the possible interaction between ATG5 and miR-30c-5p in PD. To explore this interaction, MPTP-induced PD mice and MPP+-stimulated PD-SH-SY5Y cells were constructed, and the effects of miR-30c-5p on mice behaviors and oxidative stress, apoptosis and autophagy-related pathways were examined. The possible mechanism of miR-30c-5p in PD was further analyzed. Based on the experiments above, we aimed to discover the function of miR-30c-5p in PD, hoping to provide a potential therapeutic target for PD in the future.

## Materials and Methods

### Statements

Animal experiments were approved by the Fourth Hospital of Harbin Medical University Animal Ethics Committee (No. 20181123A). Efforts were made to reduce the number of mice used and their suffering.

### Animal Model

Forty-eight male C57BL/6 mice (8 weeks, 20–22 g) were obtained from the Model Animal Research Center of Nanjing University. The mice were divided into eight groups, with six mice in each group. The stereotactic intraventricular injection site was chosen as previously reported (Hu et al., 2019). For the delivery of miR-30c-5p and agomiR, a stereotactic catheter was surgically delivered into the right lateral ventricle of mice (−2.8 mm anteroposterior, −1.2 mm mediolateral, and −4.3 mm dorsoventral). The miR-30c-5p agomiR (agomiR; miR40000514-4-5), and its negative control (agomiR-NC; miR4N0000001-4-5), miR-30c-5p antagomiR (antagomiR; miR30000514-4-5) and its negative control (antagomiR-NC; miR3N0000001-4-5) were purchased from Guangzhou Ribobio Company Limited.

Mice of the control group were given intraperitoneal injection of saline only for 2 weeks. Mice of the model group were administered intraperitoneal injection of 15 mg/kg neurotoxin MPTP for 2 weeks. Then the mice of both groups were daily given 5 μl of saline from the 14th day (2 h after MPTP treatment) to the 20th day through right lateral intracerebroventricular injection. The agomiR-NC group was daily given right lateral intracerebroventricular injection of agomiR control (20 nmol/L, 5 μl) from the 14th day to the 20st day. The agomiR group was daily given miR-30c-5p agomiR (20 nmol/L, 5 μl) from the 14th day to the 20 day through right lateral intracerebroventricular injection. The antagomiR-NC group was daily given antagomiR control (20 nmol/L, 5 μl) from the 14th day to the 20th day through right lateral intracerebroventricular injection. The antagomiR group was daily given miR-30c-5p antagomiR (20 nmol/L, 5 μl) from the 14th day to the 20th day through right lateral intracerebroventricular injection. The above injection of agomiR, agomiR-NC, antagomiR-NC or antagomiR began 2 h after the last injection of MPTP. Twenty-four hours after the last injection, the behavioral assessments were accessed and then the mice were sacrificed by decapitation.

To examine the effect of 3-MA on PD, the same operation was conducted. The antagomiR-NC+3-MA group was daily given antagomiR control (20 nmol/L, 5 μl) and 0.3 mg of 3-MA through right lateral intracerebroventricular injection from the 14th day to the 20th day, and the mice were sacrificed 24 h after the last injection. AntagomiR+3-MA group was daily given saline (5 μl) containing miR-30c-5p antagomiR (20 nmol/L) and 0.3 mg of 3-MA through right lateral intracerebroventricular injection from the 14th day to 20th day, and the mice were sacrificed by cervical dislocation 24 h after the last injection. Then the ventral midbrain containing the substantia nigra pars compacta was dissected and stored at −80°C for further experiment.

ATG5-specific siRNA (siATG5) and the negative control siRNA (siNC) were purchased from Guangzhou Ribobio Company Limited. Furthermore, siATG5 and siNC injection to the MPTP model mice were used to further explore the involvement of ATG5 and autophagy regulated/targeted by miR-30c-5p in PD. Mice were randomly divided into seven groups: control, model, antagomiR-NC, antagomiR, siNC, siATG5, antagomiR+siATG5. The protocol was conducted similar to that mentioned above.

### Quantitative Polymerase Chain Reaction

RNA was extracted from tissue homogenate or cells by Trizol and then the First-Strand Synthesis System (cat: 18091200, Invitrogen, Carlsbad, CA, USA) was used in the reverse transcription of RNA into cDNA. Next, the cDNA was subjected to qPCR using SYBR Green/ROX qPCR Master Mix (cat: K0223, Thermo Fisher Scientific, Waltham, MA, USA). The condition for the three-step PCR was set as follows: at 95°C for 15 s; at 60°C for 20 s; at 72°C for 20 s for 40 cycles. GAPDH and U6 snRNA were used as internal controls, and the relative expression was calculated through 2-^ΔΔCt^ method (Lin et al., 2018). The primer information was listed in Table 1.

**Table 1:** The primer sequences were used for qPCR in this study.

Gene	Primer sequence	Species
GAPDH	5′-AGGTCGGTGTGAACGGATTTG-3′ 5′-GGGGTCGTTGATGGCAACA-3′	Mouse
GAPDH	5′-CCACTCCTCCACCTTTGAC-3′ 5′-ACCCTGTTGCTGTAGCCA-3′	Human
Bax	5′- AGACAGGGGCCTTTTTGCTAC -3′ 5′- AATTCGCCGGAGACACTCG -3′	Mouse
Bax	5′-CACAACTCAGCGCAAACATT-3′ 5′-ACAGCCATCTCTCCATGC-3′	Human
Bcl-2	5′-GCTACCGTCGTGACTTCGC-3′ 5′-CCCCACCGAACTCAAAGAAGG-3′	Mouse
Bcl-2	5′-GAAGCACAGATGGTTGATGG-3′ 5′-CAGCCTCACAAGGTTCCAAT-3′	Human
miR-30c-5p	5′-AGCGTCGTATCCAGTGCAAT-3′ 5′-GTCGTATCCAGTGCGTGTCG-3′	Mouse
miR-30c-5p	5′-AGCGTCGTATCCAGTGCAAT-3′ 5′-GTCGTATCCAGTGCGTGTCG-3′	Human
ATG5	5′-TGTGCTTCGAGATGTGTGGTT-3′ 5′-ACCAACGTCAAATAGCTGACTC-3′	Mouse
ATG5	5′-AAAGATGTGCTTCGAGATGTGT-3′ 5′-CACTTTGTCAGTTACCAACGTCA-3′	Human
U6	5′-CTCGCTTCGGCAGCACATATACT-3′ 5′-ACGCTTCACGAATTTGCGTGTC-3′	Mouse
U6	5′-CGCTTCACGAATTTGCGTGTCAT-3′ 5′-GCTTCGGCAGCACATATACTAAAAT-3′	Human

### Behavioral Evaluation

Twenty-four hours after intracerebroventricular injection, rotarod test, grip strength test and footprinting test were performed (Singh et al., 2017). Mice from each group were pre-trained before the tests. For the rotarod test, the mice were placed into a rotarod apparatus (Harvard Apparatus, MA, USA) at a speed of 30 rpm. Then, the latencies to fall were recorded by magnetic trip plates. For the grip strength test, the forelimb grip strength of the mice was recorded using a computerized grip strength meter (TSE, Germany). For the footprinting test, mice were pre-trained to walk across a white sheet of paper without interruption. The forepaws of the mice were labeled with ink, and the distance between each step on the same side was recorded and the stride length was calculated.

### Detection of Malondialdehyde (MDA), Catalase (CAT), and SOD

Malondialdehyde (MDA) Assay Kit (cat: S0131S, Beyotime, China), Catalase Assay Kit (cat: S0051, Beyotime, China), and CuZn/Mn-SOD Assay Kit (cat: S0103, Beyotime, China) were purchased to measure the content of MDA, catalase (CAT) and SOD in tissues or cells, respectively. Briefly, for MDA detection, tissue homogenate or cell lysate was collected by centrifugation (4°C, 10,000× *g*, 10 min). The obtained supernatant was then used to determine the protein content using the BCA method (cat: P0009, Beyotime, China). Next, 100 μl of the supernatant was mixed with 200 μl of MDA working regent and heated at 100°C for 15 min. After that, the mixture was collected by centrifugation (4°C, 10,000× *g*, 10 min), and then 200 μl of supernatant was added into a 96-well plate. Finally, the absorbance was measured at 532 nm with a microreader (PLUS 384, Molecular Devices, San Jose, CA, San Jose, CA, USA). For CAT detection, 40 μl of tissue or cell supernatant containing 25–30 μl of CAT detection buffer and 10 μl of 250 mM H_2_O_2_ solution was added into a cuvette. After incubation for 5 min at 25 °C, 450 μl of stop solution was added to stop the reaction. After 15 min, the mixture was diluted by CAT detection buffer in a 96-well plate and then mixed with chromogenic agent (200 μl) for 15 min. Finally, the absorbance at 520 nm was detected using the microreader (PLUS 384, Molecular Devices, San Jose, CA, USA). For SOD detection, 20 μl of tissue or cell supernatant was mixed with WST-8 working solution (160 μl) and reaction initiating working solution (20 μl). After incubation at 37°C for 30 min, the absorbance at 450 nm was measured under a microreader (PLUS 384, Molecular Devices, USA). Each sample was set with six parallel tests.

### Western Blot

Brain tissues or cells were collected for determining protein level (Perrech et al., 2019). Tissue homogenate or cell lysate in the RIPA lysis buffer was centrifuged at 4°C (15,000× *g*, 15 min). The protein content was detected using BCA (Thermo Fisher Scientific, Waltham, MA, USA). Then, 30 μg of protein was separated by 12% SDS-PAGE and then transferred to PVDF (Invitrogen, USA). After the membranes were blocked with 5% skimmed milk for 2 h at room temperature, the following primary antibodies were used to induce an immunoreaction with the protein on the membranes: Bcl-2 (ab182858, 1:2,000 dilution, Abcam, Cambridge, MA, USA); Bax (ab32503, 1:1,000 dilution, Abcam); cleaved-caspase-3 (ab49822, 1:500 dilution, Abcam); GAPDH (ab181602, 1:10,000 dilution, Abcam); p62 (ab109012, 1:10,000 dilution, Abcam); LC3 II /LC3 I (ab48394, 1:1,000 dilution, Abcam); ATG5 (ab108327, 1:1,000 dilution, Abcam). Afterward, HRP-coupled IgG H&L (ab205718, 1:2,000 dilution, Abcam, Cambridge, MA, USA) as the secondary antibody was incubated with the membrane. Then, the antibody-bound protein blots were developed with an enhanced chemiluminescence (ECL) kit (P0018S, Beyotime, Shanghai, China). Finally, ImageJ 1.48v (National Institutes of Health, USA) was used to quantify the blot intensities. GAPDH served as an internal control.

### Detection of Dopamine Metabolic Markers

HPLC analysis was performed to determine the content of dopamine, dihydroxyphenylacetic acid (DOPAC), and homovanillic acid (HVA) in substantia nigra tissues using a Diode Array Detector. Briefly, brain tissues were immersed in PBS and homogenized using a tissue homogenizer. After centrifugation at 10,000× *g*, 4°C for 20 min, the supernatant (20 μl) was collected and delivered into a HPLC system. Then the flow rate of the mobile phase containing buffer acetate (pH 4.66) and methanol (pH 4.2) was adjusted to 0.8 ml/min, and the column temperature was maintained at 40°C. Finally, absorbance at the wavelengths for dopamine (220 nm), DOPAC (260 nm), and HVA (280 nm) was recorded, and Empower software was used to conduct the HPLC process and collect data.

### Immunohistofluorescence

Mouse brain tissues were separated and fixed with 4% paraformaldehyde for 24 h at room temperature. Then the tissues were prepared into paraffin sections after dehydration, wax dip, and embedding. Next, the sections were heated at 60°C for 20 min, and then subjected to dewaxing, hydration, and antigen retrieval in sodium citrate buffer solution. Afterward, the sections were blocked with serum and then incubated with anti-Tyrosine hydroxylase antibody (1:100, cat: ab113, Abcam) at 4°C overnight. Subsequently, DyLight 594-conjugated donkey anti-sheep IgG (cat: ab96941, Cambridge, MA, USA) was incubated with the sections for 1 h at room temperature as the secondary antibody. Finally, the tyrosine hydroxylase (TH) staining in the Substantia nigra pars compacta (SNpc) was estimated using the fluorescence intensity by a Leica inverted fluorescence microscopy (DM2500, Leica, German). A relative TH level of the test group was compared with the control value set at 1.

### SH-SY5Y Culture and MPP+ Treatment

SH-SY5Y cells (cat: CRL-2266) were purchased from ATCC (USA) and grown in DMEM (cat: 11965092, Gibco, Grand Island, NY, USA) supplemented with 15% FBS (cat: 12483020, Gibco), penicillin (100 U/ml), and streptomycin (100 μg/ml, cat: 10378016, Gibco) at 37°C. 1-methyl-4-phenylpyridinium (MPP+, cat: D048) was purchased from Sigma (St. Louis, MO, USA). To induce a PD *in vitro* model, SH-SY5Y cells were incubated with MPP+ (5 mM) for 18 h at 37°C.

### Cell Transfection

Before MPP+ treatment, the cells were pre-transfected with inhibitor control (IC, AAGUUCUUCUCCUUUACUCAU), inhibitor (I, 5’-GCUGAGAGUGUAGGAUGUUUACA-3’), negative control (NC, sense: 5’-AAGUUCUUCUCCUUUACUCAU-3’; antisense: 5’- GAGUAAAGGAGAAGAACUUUU-3’) or siATG5 (sense: 5’-AAACAAGUUGGAAUUCGUCCA-3’; antisense: 5’- GACGAAUUCCAACUUGUUUCA-3’); siNC (sense, 5′-UUCUCCGAACGUGUCACGUTT-3′ and antisense, 5′-ACGUGACACGUUCGGAGAATT-3′).

### Dual-Luciferase Reporter Assay

A 96-well plate for chemiluminescence detection was used for cell culture and further luciferase detection. The recombinant plasmid pmirGLO containing the 3’UTRs of ATG5-WT and ATG5-MUT was constructed. Then, SH-SY5Y cells were transfected with ATG5-WT or ATG5-MUT reporter plasmid in the presence of inhibitor control or miR-30c-5p inhibitor using Lipofectamine 2000 (cat: 11668019, Carlsbad, CA, USA). After 24 h of culture, the cells were lysed, and then the firefly luciferase activity and the renilla luciferase activity were separately detected using a dual-luciferase assay system (Promega, Madison, WI, USA) and a luminometer (cat: 11300010, Berthold, Germany).

### MTT Assay

The cells (2 × 10^3^ cells/well) were seeded into a 96-well plate. After 24 h, 48 h or 72 h of culture, cell viability was detected using MTT reagent (cat: C0009, Beyotime, China). After incubating the cells with MTT at 37°C for 4 h, formazan reagent (100 μl) was added into each well to dissolve the purple crystal. Then, the absorbance at 570 nm was measured under a microplate reader (PLUS 384; Molecular Devices, USA).

### Flow Cytometry

An Annexin V-FITC kit (cat: C1062S, Beyotime, China) was purchased for apoptosis analysis. Briefly, the cells were digested using 0.25% trypsin and then centrifuged at 500× *g*, 4°C for 5 min. After the centrifugation, the cells were collected and washed with binding buffer. Subsequently, the cells were labeled with 5 μl of Annexin-V-FITC (provided in the kit), followed by reacting with 10 μl PI (provided in the kit). After incubation for 15 min at room temperature, the cells were cooled on ice. Finally, the fluorescence intensity was determined using a flow cytometer (cat: 342973, BD Biosciences, USA), and the apoptosis rate was analyzed using the FACSCanto^TM^ system software v2.4 (cat: 646602, BD Biosciences, USA).

### Statistical Analysis

All the data were represented as mean ± SD. Differences between the two groups were analyzed using the Student’s *t- test*. Differences among groups were analyzed using one-way or two-way analysis of variance (ANOVA) followed by Tukey’s test. *P* < 0.05 indicated that the difference was statistically significant. Statistical data (F, degrees of freedom, and significance for the factors and interactions) are shown in Supplementary Table 1.

## Results

### Overexpression or Inhibition of miR-30c-5p in MPTP-Induced PD Mice

**Figure 1:**
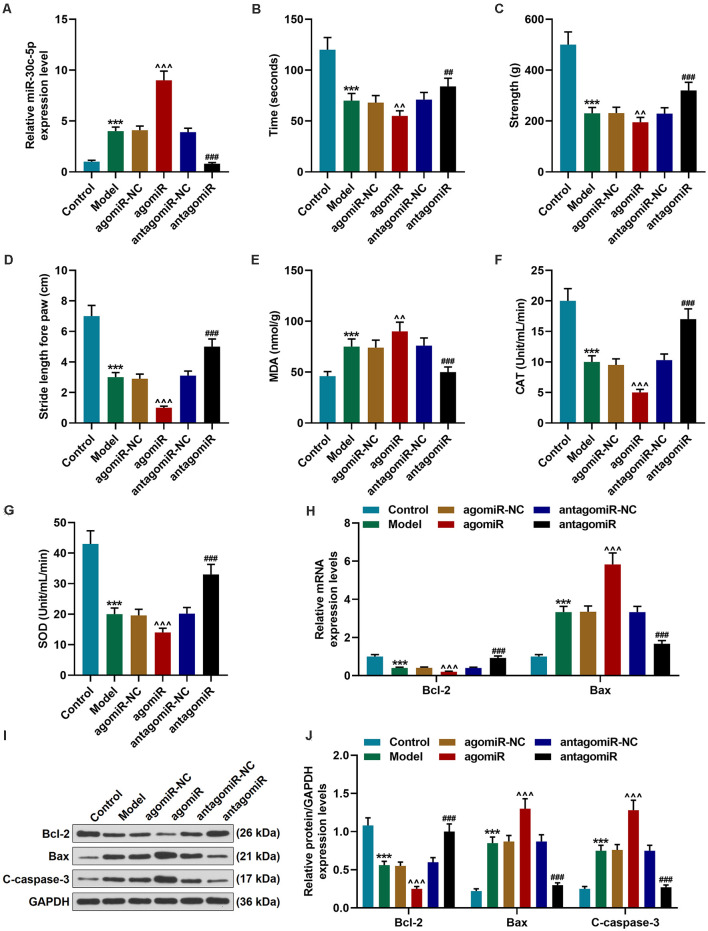
Effect of miR-30c-5p on behavioral symptoms and the levels of oxidative stress-related factors and apoptosis-related proteins in Parkinson’s disease (PD) mice. **(A)** QPCR analysis of the transfection efficiency of miR-30c-3p agomiR or antagomiR in PD mice brain tissues. U6 snRNA was served as an internal control. **(B)** The time that the mice stayed on the rotarod was recorded. **(C)** The grip strength test was used to measure the forelimb grip strength. **(D)** The locomotor function was assessed using the footprinting test. **(E–G)** Oxidative stress-related factors malondialdehyde (MDA), catalase (CAT), and SOD were detected in mice. **(H)** Expressions of Bcl-2 and Bax in brain tissues were determined by qPCR. **(I,J)** Expressions of Bcl-2, Bax and, cleaved-caspase-3 (C-caspase-3) in brain tissues were assessed by Western blot. GAPDH served as an internal control. ***p < 0.001 vs. control group. ^∧∧^*p* < 0.01, ^∧∧∧^*p* < 0.001 vs. agomiR-NC group. ^##^*p* < 0.01, ^###^*p* < 0.001 vs. antagomiR-NC group.

Recently, miR-30-5p has been reported to be significantly upregulated in PD patients (Vallelunga et al., 2019). To further investigate the role of miR-30-5p *in vivo*, miR-30c-5p agomiR or antagomiR was transfected into MPTP-induced PD mice. The result showed that miR-30-5p was obviously upregulated in PD mice (Figure 1A; *p* < 0.001), and as expected, miR-30-5p expression was obviously promoted in the agomiR group (Figure 1A; *p* < 0.001). It was also noted that miR-30-5p was suppressed in the antagomiR group (Figure 1A; *p* < 0.001).

Next, we performed behavioral tests to evaluate the motor function of the mice. The rotarod test is widely used to assess the motor coordination in animals through monitoring the latency to fall from the rotary rod. Previous reports have shown that MPTP could induce rotarod behavioral decline (Ayton et al., 2013). Our results demonstrated that there was no remarkable difference in the time that the animals stayed on the rotarod between the agomiR-NC group and Model group. The time that the mice stayed on the rotarod was significantly reduced in the agomiR group, as compared with the agomiR-NC group (Figure 1B; *p* < 0.01), however, in the antagomiR group, the time that the mice stayed on the rotarod was markedly increased, as compared with the antagomiR-NC group (Figure 1B; *p* < 0.01). MPTP treatment significantly reduced the forelimb grip strength of the mice, as compared with the control group (Figure 1C; *p* < 0.001), and miR-30c-5p agomiR remarkably reduced the forelimb grip strength of the mice (Figure 1C; *p* < 0.01). At the same time, as expected, the forelimb grip strength of the mice in antagomiR group was significantly increased, as compared with that in the antagomiR-NC group (Figure 1C; *p* < 0.001). The footprinting test was performed to assess the locomotor ability of the mice. As shown in Figure 1D, the stride length of the mice was significantly shortened in the Model group (Figure 1D; *p* < 0.001), and greatly reduced in the agomiR group, as compared with the agomiR-NC group (Figure 1D; *p* < 0.001), moreover, antagomiR could noticeably increase the stride length of mice, as compared with the antagomiR-NC group (Figure 1D; *p* < 0.001).

The levels of oxidative stress-related factors were detected using commercial kits. MDA is a widely accepted marker for oxidative stress. It was found that MDA level was markedly upregulated in the Model group (Figure 1E; *p* < 0.001), and agomiR treatment further upregulated the level of MDA (Figure 1E; *p* < 0.01), however, miR-30c-5p antagomiR obviously downregulated the MDA level (Figure 1E; *p* < 0.001). Meanwhile, CAT and SOD levels were significantly downregulated in the Model group (Figures 1F,G; *p* < 0.001), and agomiR further downregulated the levels of CAT and SOD (Figures 1F,G; *p* < 0.001), however, CAT and SOD levels were remarkably upregulated in the antagomiR group, as compared with the antagomiR-NC group (Figures 1F,G; *p* < 0.001).

The expression levels of apoptosis-associated proteins were further detected by qPCR and Western blot. Bcl-2, an anti-apoptotic protein, showed markedly downregulated expression in the Model and agomiR groups (Figures 1H–J; *p* < 0.001), while miR-30c-5p antagomiR obviously upregulated the expression of Bcl-2 (Figures 1H–J; *p* < 0.001). In addition, the pro-apoptotic protein Bax and the apoptotic marker cleaved-caspase-3 (C-caspase-3) expressions were significantly upregulated in the Model and agomiR groups, whereas miR-30c-5p antagomiR obviously downregulated Bax and caspase-3 levels (Figures 1H–J; *p* < 0.001). The data indicated that miR-30c-5p downregulation attenuated apoptosis in brain tissues of MPTP-treated mice.

### MiR-30c-5p Decreased the Content of Dopamine and Its Metabolites and Inhibited the Activation of Autophagy

**Figure 2:**
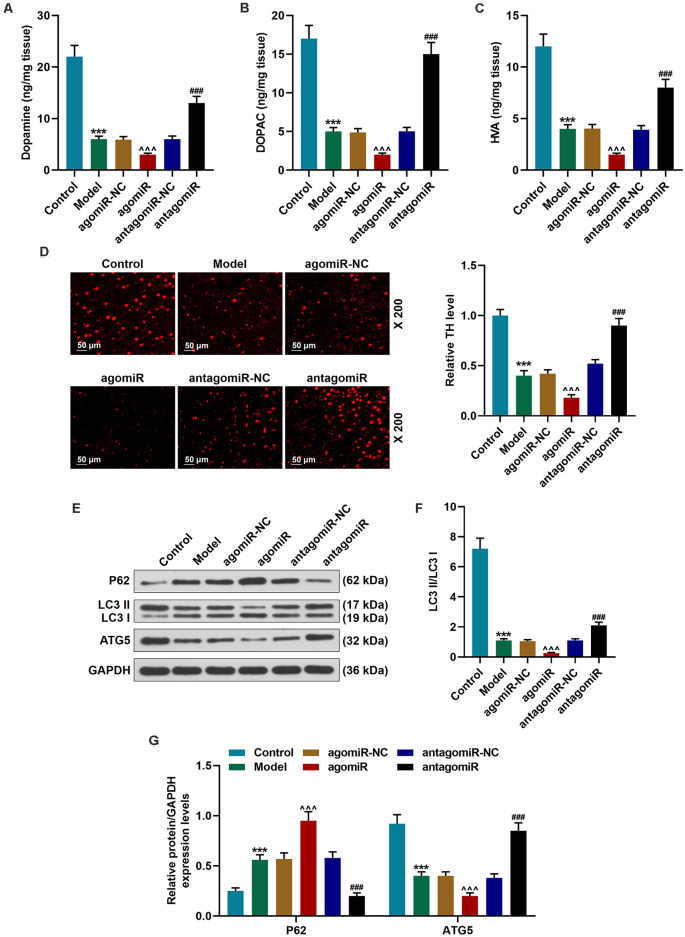
Detection of dopamine metabolic markers, tyrosine hydroxylase (TH), and autophagy pathway-associated proteins in PD mice after transfection of miR-30c-5p overexpression or knockdown. **(A–C)** Dopamine metabolic markers dopamine, dihydroxyphenylacetic acid (DOPAC), and homovanillic acid (HVA) were detected using Diode Array Detector (DAD). **(D)** The level of TH in the Substantia nigra pars compacta (SNpc) was determined by immunofluorescence. Scale bar = 50 μm. 200×. **(E–G)** Western blot analysis of the expressions of autophagy-related proteins (p62, LC3 II, and LC3 I, ATG5) in brain tissues. The ratio of LC3 II to LC3 I was calculated. GAPDH served as an internal control. ***p < 0.001 vs. control group. ^∧∧∧^*p* < 0.001 vs. agomiR NC group. ^###^*p* < 0.001 vs. antagomiR NC group.

MPP+ is selectively transported into nigra neurons *via* the mesencephalic dopamine transporter (DAT) and selectively destroys dopamine neurons in the substantia nigra. Our data indicated that the levels of dopamine and its metabolites were significantly downregulated in the Model group (Figures 2A–C; *p* < 0.001). In the antagomiR group, however, compared with the antagomiR-NC group, the levels of dopamine, the expressions of DOPAC and HVA were obviously upregulated (Figures 2A–C; *p* < 0.001). TH is a critical enzyme for the synthesis of dopamine. Immunofluorescence staining results showed that TH level in the SNpc of the Model group and the agomiR group was obviously reduced (Figure 2D). In the antagomiR group, however, TH level was increased, as compared with the antagomiR-NC group (Figure 2D). Autophagy mitigates the death of dopaminergic neuronal cells by removing damaged or dysfunctional protein and organelles (Giordano et al., 2014). Previous research demonstrated that autophagy is inhibited in MPTP-treated mice (Sun et al., 2018). The protein level of P62 was upregulated and the LC3II/LC3I ratio and ATG5 expression were reduced and downregulated in the Model group (Figures 2E–G; *p* < 0.001), moreover, agomiR further promoted the effect (Figures 2E–G; *p* < 0.001), while antagomiR reversed the effect in brain tissues of MPTP-treated mice (Figures 2E–G; *p* < 0.001).

### MiR-30c-5p Regulated the Activation of Autophagy in Dopaminergic Neurons

**Figure 3:**
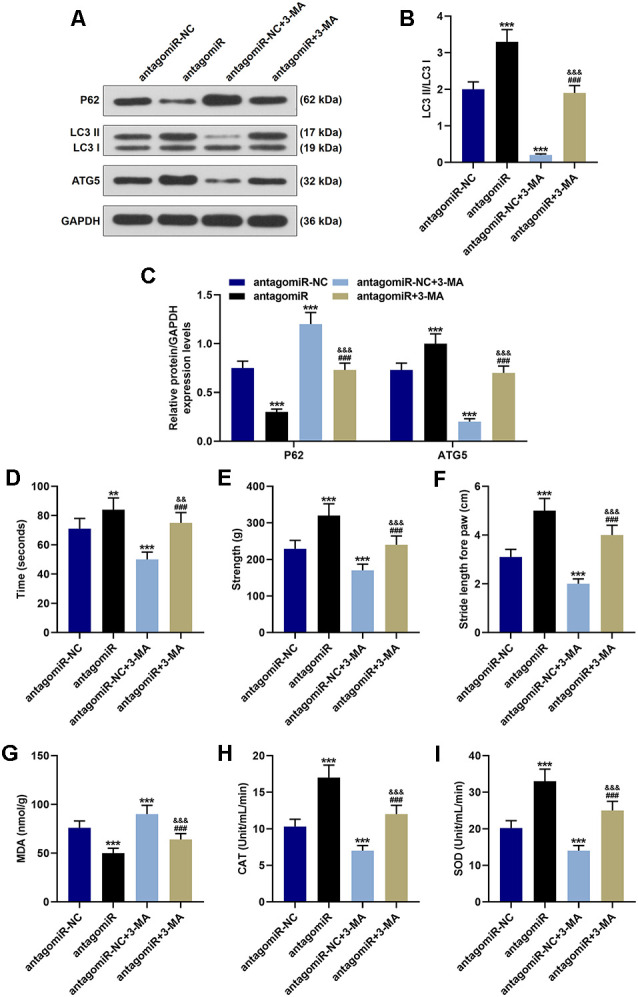
Effect of miR-30c-5p on behavioral symptoms and oxidative stress level in PD mice through autophagy. **(A–C)** Western blot analysis of autophagy-related proteins (p62, LC3 II, LC3 I, and ATG5) in brain tissues of mice. The ratio of LC3 II to LC3 I was calculated. **(D)** The time that the mice stayed on the rotarod was recorded. **(E)** The grip strength test was used to measure the forelimb grip strength. **(F)** The locomotor function was assessed using the footprinting test. **(G–I)** Oxidative stress-related factor levels, MDA, CAT, and SOD were detected in mice. **p < 0.01, ***p < 0.001 vs. antagomiR-NC. ^###^*p* < 0.001 vs. antagomiR-NC +3-MA. ^&&^*p* < 0.01, ^&&&^*p* < 0.001 vs. antagomiR.

3-Methyladenine (3-MA) is commonly used as the inhibitor of autophagy. Our results indicated that antagomiR significantly promoted the activation of autophagy, which was reflected by the increase of LC3 II/LC3 I ratio and ATG5 as well as the decrease of p62 when compared with the antagomiR-NC group (Figures 3A–C; *p* < 0.001). In the antagomiR-NC + 3-MA group, autophagy was inhibited compared with the antagomiR-NC group (Figures 3A–C; *p* < 0.001). The activation of autophagy was obviously rescued in the antagomiR+3-MA group compared with the antagomiR group (Figures 3A–C; *p* < 0.001). The time that the animals stayed on the rotary rod was significantly reduced after 3-MA treatment (Figure 3D; *p* < 0.001), meanwhile, the grip strength and the stride length of mice were markedly decreased after the injection of 3-MA (Figures 3E,F; *p* < 0.001). Moreover, 3-MA treatment attenuated the effect of antagomiR on promoting the time that the animals stayed on the rotary rod, the grip strength, and the stride length of mice (Figures 3D–F; *p* < 0.01). Furthermore, oxidative stress-related factors (MDA, CAT, and SOD) were detected, and the results showed that the oxidative stress level in the antagomiR group was obviously lower than that in the antagomiR-NC group, as MDA was decreased and CAT and SOD were increased, while 3-MA had an opposite effect on cell oxidative stress when compared with antagomir group, as MDA was increased and CAT and SOD were decreased (Figures 3G–I). Moreover, 3-MA treatment attenuated the effect of antagomiR on oxidative stress-related factors MDA, CAT, and SOD (Figures 3G–I; *p* < 0.001).

### MiR-30c-5p Regulated Dopaminergic Neuronal Apoptosis and Dopamine Metabolites by Modulating Autophagy

**Figure 4:**
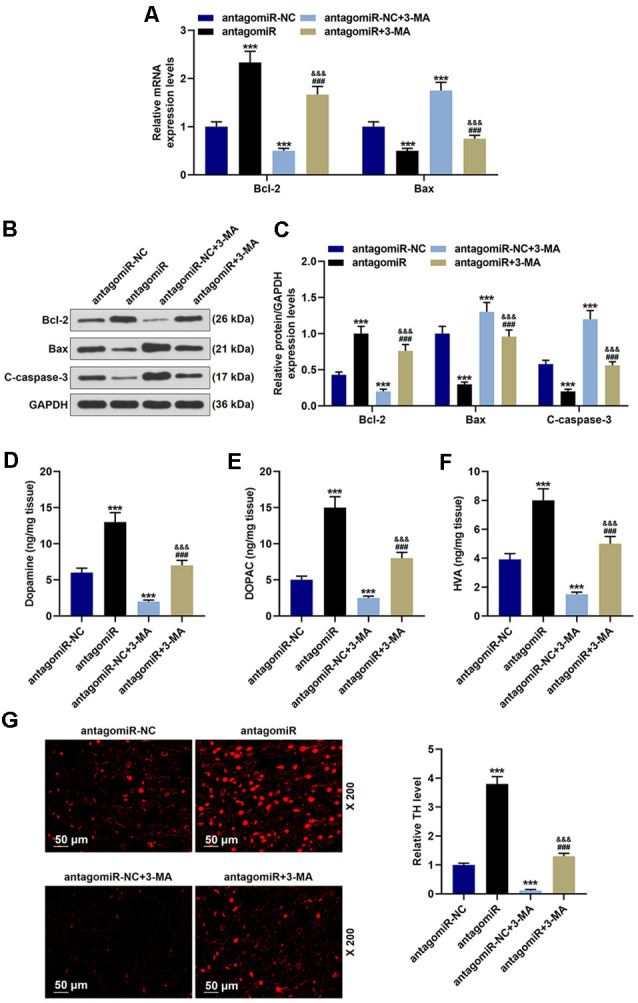
Effects of miR-30c-5p and autophagy on apoptosis-related proteins, dopamine metabolic markers, and TH in PD mice. **(A)** Expressions of Bcl-2 and Bax were determined by qPCR. **(B,C)** Expressions of Bcl-2, Bax, and cleaved-caspase-3 (C-caspase-3) in brain tissues were assessed by Western blot. GAPDH served as an internal control. **(D–F)** Dopamine metabolic markers dopamine, DOPAC, and HVA in mice were detected using a Diode Array Detector. **(G)** The level of TH in the SNpc was determined by immunofluorescence. Scale bar = 50 μm. 200×. ***p < 0.001 vs. antagomiR-NC. ^###^*p* < 0.001 vs. antagomiR-NC +3-MA. ^&&&^*p* < 0.001 vs. antagomiR.

Further experiments indicated that antagomiR also alleviated apoptosis in the MPTP-induce PD mice model through modulating autophagy. As shown in Figures 4A–C, antagomiR treatment induced the increase of Bcl-2 and the decrease of Bax and C-caspase-3 (Figures 4A–C; *p* < 0.001), however, after 3-MA treatment, the apoptosis was markedly reduced, with the increase of Bax and C caspase-3 and the reduction of Bcl-2 (Figures 4A–C; *p* < 0.001). Moreover, 3-MA treatment attenuated the effect of antagomiR on apoptosis-related molecules Bcl-2 Bax and C-caspase-3 (Figures 3G–I; *p* < 0.001). Dopamine and its metabolites were detected using a Diode Array Detector (DAD), and we observed that antagomiR could significantly increase dopamine and its metabolites (Figures 4D–F; *p* < 0.001), but its effect was moderately alleviated by 3-MA (Figures 4D–F; *p* < 0.001). Moreover, the promoting effect of antagomiR on TH level in the SNpc was also alleviated by 3-MA (Figure 4G).

### ATG5 Was a Target of MiR-30c-5p

**Figure 5:**
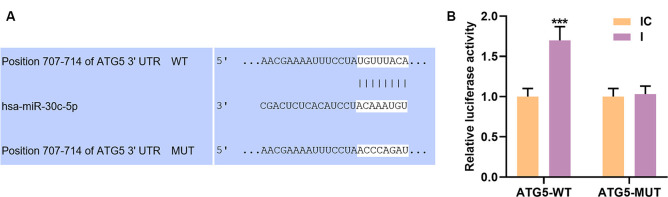
Autophagy-related gene 5 protein (ATG5) was a target gene of miR-30c-5p. **(A)** The potential binding site of miR-30c-5p on the 3’UTR of ATG5 was obtained using TargetScan. **(B)** Dual-luciferase reporter assay was performed on SH-SY5Y cells to assess the targeting relationship between ATG5 and miR-30c-5p. IC: inhibitor control. I: miR-30c-5p inhibitor. ***p < 0.001 vs. IC.

TargetScan V7.2 is a public target database. In this study, targetScan V7.2 predicted that miR-30c-5p had a binding site on ATG5 3’-UTR (Figure 5A). To verify this prediction, we cloned the binding site into a dual-luciferase reporter plasmid, which was subsequently co-transfected into cells with miR-30c-5p inhibitor (I) or IC. We found that miR-30c-5p inhibitor significantly enhanced the activity of luciferase reporter constructs containing ATG5-WT (Figure 5B; *p* < 0.001), while no obvious change was observed in the luciferase activity in the cells transfected with ATG5 3’UTR mutation constructs (Figure 5B).

### MiR-30c-5p Promoted the Progression of PD by Targeting ATG5 *In vitro*

**Figure 6:**
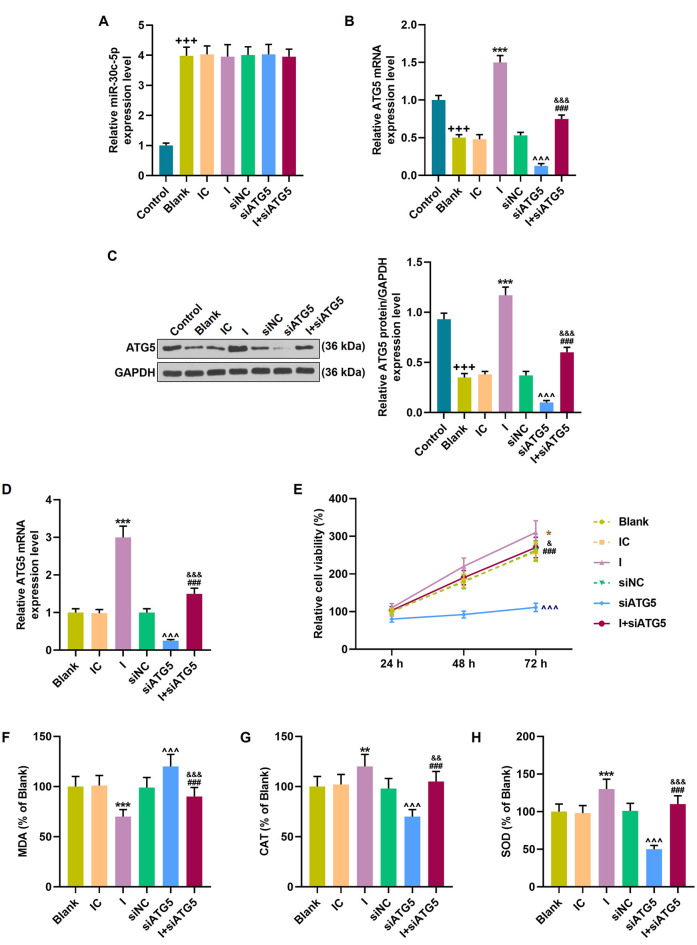
MiR-30c-5p inhibitor regulated cell viability and the levels of oxidative stress-related factors in MPP+ stimulated SH-SY5Y cells *via* ATG5. **(A)** qPCR analysis of miR-30c-5p expression in MPP+ induced PD cells. U6 snRNA served as an internal control. **(B–D)** ATG5 expression in SH-SY5Y cells was detected by Western blot. GAPDH served as an internal control. **(E)** The viability of SH-SY5Y cells was measured by MTT assay. **(F–H)** The levels of oxidative stress-related factors MDA, CAT, and SOD were detected. ^+++^*p* < 0.001 vs. control. *p < 0.05, **p < 0.01, ***p < 0.001 vs. IC. ^∧∧∧^*p* < 0.001 vs. NC. ^&^*p* < 0.05, ^&&^*p* < 0.01, ^&&&^*p* < 0.001 vs. I. ^###^*p* < 0.001 vs. siATG5. NC, negative control for siRNA.

To further explore the interaction between miR-30c-5p and AGT5, miR-30c-5p inhibitor and siATG5 were transfected into SH-SY5Y cells. Compared to the control group (untreated SH-SY5Y cells), the miR-30c-5p level was increased in the blank group (MPP+ stimulated SH-SY5Y cells), while AGT5 level was increased in the blank group (MPP+ SH-SY5Y cells; Figures 6A,B; *p* < 0.001). However, silencing of AGT5 did not affect miR-30c-5p expression (Figure 6A), but miR-30c-5p downregulation elevated the level of AGT5 (Figures 6B–D; *p* < 0.001), and siATG5 attenuated the effect of miR-30c-5p inhibitor on promoting AGT5 expression in MPP+ SH-SY5Y cells (Figures 6A–D; *p* < 0.001). MTT assay results demonstrated that miR-30c-5p downregulation promoted cell viability, while siATG5 showed the opposite effect. Moreover, co-treatment with miR-30c-5p inhibitor and siATG5 reversed the effect of treatment with miR-30c-5p inhibitor or siATG5 alone (Figure 6E). The oxidative stress level of the cells was reduced by miR-30c-5p inhibitor, with the decrease of MDA and the increase of CAT and SOD, while these effects were attenuated by siATG5 (Figures 6F–H; *p* < 0.001).

**Figure 7:**
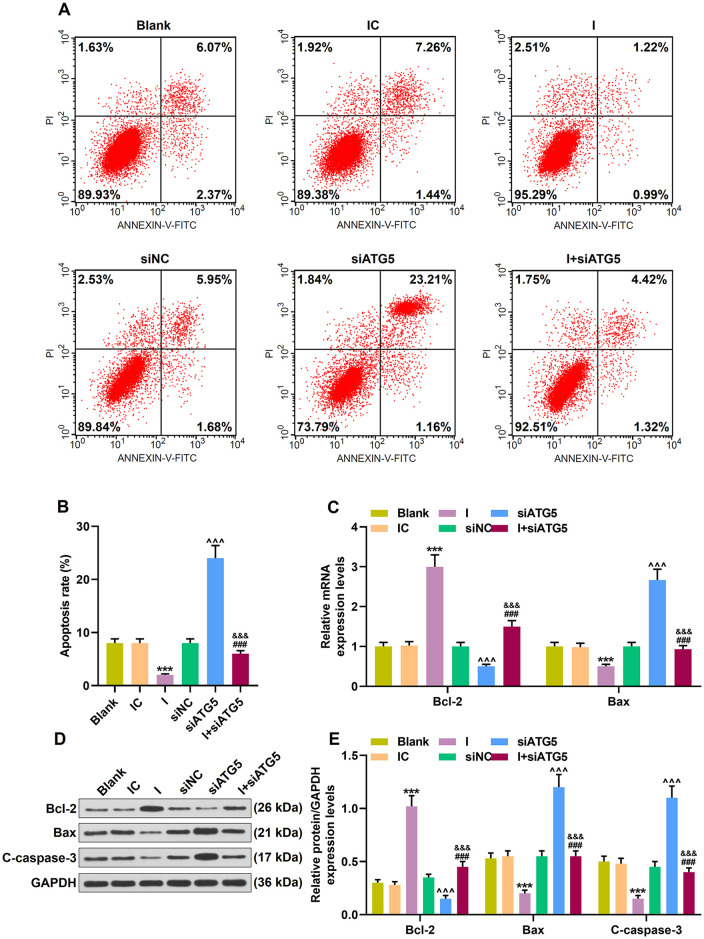
Effect of MiR-30c-5p inhibitor on cell apoptosis and the expressions of apoptosis-related proteins in MPP+ stimulated SH-SY5Y cells *via* ATG5. **(A)** Flow cytometry analysis of the apoptosis rate of cells. **(B)** The apoptosis rate was calculated. **(C)** Expressions of Bcl-2 and Bax were determined by qPCR. **(D,E)** Expressions of Bcl-2, Bax and, cleaved caspase-3 (C-caspase-3) in SH-SY5Y cells were assessed by Western blot. GAPDH served as an internal control. ***p < 0.001 vs. IC. ^∧∧∧^*p* < 0.001 vs. NC. ^&&&^*p* < 0.001 vs. I. ^###^*p* < 0.001 vs. siATG5. NC, negative control for siRNA.

In addition, miR-30c-5p inhibitor decreased the apoptotic cells (Figures 7A,B; *p* < 0.001). However, siRNA knockdown ATG5 obviously promoted cell apoptosis (Figures 7A,B; *p* < 0.001), and miR-30c-5p inhibitor evidently alleviated the increased apoptosis rate caused by siATG5 (Figures 7A,B; *p* < 0.001). The anti-apoptosis factor Bcl-2 was markedly overexpressed, while the expressions of apoptosis-associate proteins Bax and C-caspase-3 were inhibited after the transfection of miR-30c-5p inhibitor (Figures 7C–E; *p* < 0.001). More importantly, knockdown of ATG5 showed an opposite effect of miR-30c-5p inhibitor on these apoptosis-associated markers, and siATG5 could block the effect of miR-30c-5p inhibitor on these apoptosis-associated markers (Figures 7C–E; *p* < 0.001).

### MiR-30c-5p Downregulation Attenuated the Progression of PD by Targeting ATG5 *In vivo*

**Figure 8:**
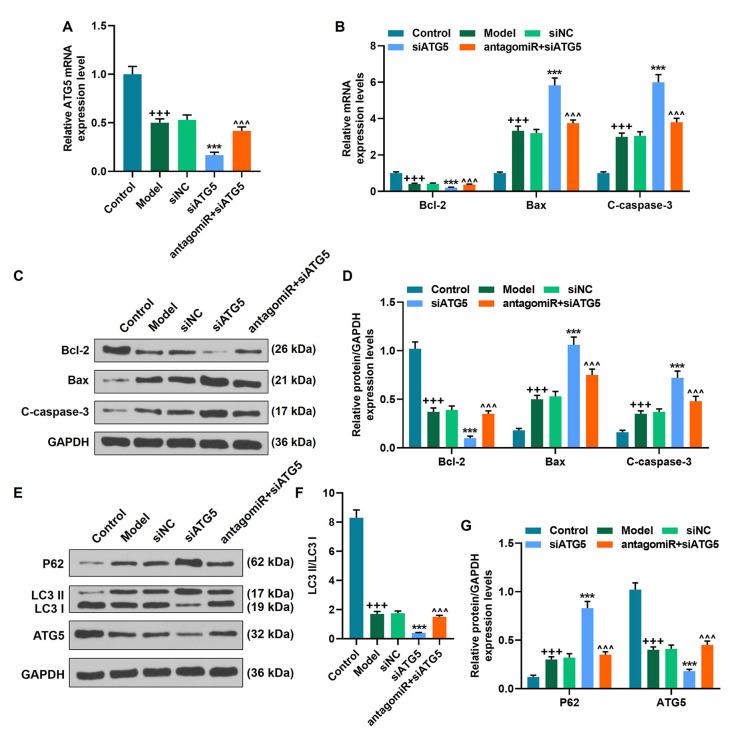
MiR-30c-5p downregulation attenuated the progression of PD by targeting ATG5 *in vivo*. **(A)** The level of ATG5 was determined by qPCR in mice. **(B–G)** The level of Bax, C-caspase-3, Bcl-2, P62 LC3 II/I, and ATG5 was determined by qPCR and Western blot. ^+++^*p* < 0.001 vs. Control. ***p < 0.001 vs. siNC. ^∧∧∧^*p* < 0.001 vs. siATG5. siNC, negative control for siRNA.

We found that ATG5 level was decreased in the Mmodel group compared to the control group, which was further promoted by siATG5 treatment; by contrast, miR-30c-5p downregulation abrogated the inhibitive effect of siATG5 on ATG5 expression in model mice (Figure 8A; *p* < 0.001). Additionally, the levels of Bax, C-caspase-3, and P62 were increased in the model group compared to the control group, while the level of Bcl-2, LC3 II/I, and ATG5 were decreased; these alters were further promoted by siATG5 treatment; by contrast, miR-30c-5p downregulation abrogated the effect of siATG5 on the levels of Bax, C-caspase-3, and Bcl-2 in model mice (Figures 8A–G; *p* < 0.001). The data suggested that miR-30c-5p antagomiR attenuated apoptosis and induced autophagy in brain tissues of MPTP-treated mice *in vivo* by upregulating ATG5 expression.

## Discussion

Parkinson’s disease (PD) is a neurodegenerative disorder characterized by progressive degeneration of dopaminergic neurons within the SNpc (Morales et al., 2015). In many neurodegenerative diseases, miRNAs are considered as reliable biomarkers for diagnosis and monitoring of disease progression. As reported in a past study, miR-30c-5p is upregulated in PD patients (Vallelunga et al., 2019). In this research, the MPTP-induced PD mice model was constructed, and miR-30c-5p expression was found to be significantly upregulated in PD. Furthermore, we discovered a link between miR-30c-5p and ATG5, and that miR-30c-5p aggravated neuronal damage *via* negatively regulating ATG5 expression and decreasing autophagy levels in the PD mice and cell model.

MPTP can penetrate the blood–brain barrier and cause selective destruction of nigrostriatal dopamine neurons in humans and animals, and it is considered to be the most valuable neurotoxin for inducing PD model in animals (Wichmann and DeLong, 2009). In this study, intraperitoneal injection of MPTP was applied to stimulate dopaminergic neuronal loss to induce PD *in vivo*. After 2 weeks of injection of MPTP, the motor deficits were significantly impaired. It was known that oxidative stress contributes to the development of PD (Hwang, 2013). In this study, the ROS indicators (MDA, CAT, and SOD) and Bcl-2, Bax, and caspase-3 were significantly dysregulated in MPTP-treated mice, which were similar to the findings of the previous research (Zhang et al., 2020). Brain mitochondrial dysfunction caused by dopamine and its major metabolites is one of the etiologies of PD (Gluck and Zeevalk, 2004). We found that dopamine and its metabolites DOAPC and HVA were significantly suppressed. TH-immunofluorescence is a characteristic of dopamine neurons (Hamanaka et al., 2016). Our result confirmed that TH levels were greatly reduced in PD mice, suggesting that dopamine neurons were damaged in PD model. Autophagy deregulation plays a critical role in PD (Moors et al., 2017), and in our research, autophagy was inhibited in PD. Taken together, the above results suggested that the PD mice model was successfully constructed.

Studies showed that miR-30c-5p plays a critical role in multiple diseases, including in atherosclerosis (Ceolotto et al., 2017), gastric cancer (Cao et al., 2017), and neuropathic pain (Tramullas et al., 2018). However, the exact role of miR-30c-5p in PD remained unknown. The present study found that overexpressed miR-30c-5p aggravated the motor deficits, increased the ROS and apoptosis, and as expected, inhibited the dopamine and autophagy levels in the PD model, suggesting that miR-30c-5p was involved in the development of PD.

Re-activation of autophagy is regarded as a promising strategy for treating neurodegenerative diseases due to its protective effect of autophagy on cells (Karabiyik et al., 2017). In this study, we noted that autophagy could be activated by miR-30c-5p antagomiR in the PD mice and that such an activation was blocked by the autophagy inhibitor 3-MA. Further analysis showed that miR-30c-5p antagomiR protected mice against PD-induced neuronic injury through regulating autophagy- and apoptosis-related pathways. Autophagy is a response to the degradation of damaged cellular organelles and proteins, while apoptosis is a response to the removal of damaged or aged cells (Liu et al., 2019). A balance between cell apoptosis and autophagy is crucial for neurons survival (Wnuk and Kajta, 2017). In this study, we observed that autophagy was suppressed and cell apoptosis was promoted in the PD model, suggesting that there was an imbalance between autophagy and apoptosis in the brain of PD mice. Further study demonstrated that ATG5, an autophagy-related gene, was the target of miR-30c-5p, indicating that miR-30c-5p played a part in PD through regulating ATG5. It was reported that the main reason for the loss of dopaminergic neurons is oxidative stress (Ghosh et al., 2009), and we also found that miR-30c-5p antagomiR promoted the survival of dopaminergic neurons through increasing TH levels *in vivo*, promoting cell viability *in vitro*, and reducing ROS level and apoptosis under the mediation of the activation of autophagy. Besides, miR-30c-5p antagomiR attenuated apoptosis and induced autophagy in brain tissues of MPTP-treated mice *in vivo* by upregulating ATG5 expression. However, it remained unknown whether ATG5 was the main target of miR-30c-5p in PD. Previous research showed that immune responses and pyroptosis, which are both involved in the progression of PD, can also be modulated by miR-30c-5p (Li et al., 2018; Zhang et al., 2019a), yet further research is needed to fully determine the role of miR-30c-5p in PD.

In this study, the levels of dopamine and its metabolites, autophagy, and neurobehavioral parameters were reduced, while ROS and apoptosis levels were increased in PD mice. Our study indicated that miR-30c-5p overexpression significantly aggravated the neurotoxicity in the PD model, while miR-30c-5p antagomiR could alleviate the damage of dopaminergic neurons through upregulating ATG5 expression. Our results provide a novel perspective of miR-30c-5p in PD and a potential target for PD treatment in the future.

## Data Availability Statement

The original contributions presented in the study are included in the article/Supplementary Material, further inquiries can be directed to the corresponding author.

## Ethics Statement

The animal study was reviewed and approved and animal experiments were approved by the The Fourth Hospital of Harbin Medical University Animal Ethics Committee (No. 20181123A).

## Author Contributions

LZ and XC: substantial contributions to conception and design, drafting the article or critically revising it for important intellectual content. MC and BJ: data acquisition, data analysis and interpretation. All authors: final approval of the version to be published, agreement to be accountable for all aspects of the work in ensuring that questions related to the accuracy or integrity of the work are appropriately investigated and resolved. All authors contributed to the article and approved the submitted version.

## Conflict of Interest

The authors declare that the research was conducted in the absence of any commercial or financial relationships that could be construed as a potential conflict of interest.

## Supplementary Material

SUPPLEMENTARY TABLE 1 Statistical data (F, degrees of freedom, and significance for the factors and interactions) were shown in supplemental spreadsheet 1.
